# Plasma membrane integrity in health and disease: significance and therapeutic potential

**DOI:** 10.1038/s41421-020-00233-2

**Published:** 2021-01-19

**Authors:** Catarina Dias, Jesper Nylandsted

**Affiliations:** 1 Membrane Integrity, Cell Death and Metabolism, Center for Autophagy, Recycling and Disease, Danish Cancer Society Research Center, Strandboulevarden 49, DK-2100 Copenhagen, Denmark; 2 Department of Cellular and Molecular Medicine, Faculty of Health Sciences, University of Copenhagen, DK-2200 Copenhagen N, Denmark

**Keywords:** Apoptosis, Mechanisms of disease, Membrane curvature, Cancer microenvironment

## Abstract

Maintenance of plasma membrane integrity is essential for normal cell viability and function. Thus, robust membrane repair mechanisms have evolved to counteract the eminent threat of a torn plasma membrane. Different repair mechanisms and the bio-physical parameters required for efficient repair are now emerging from different research groups. However, less is known about when these mechanisms come into play. This review focuses on the existence of membrane disruptions and repair mechanisms in both physiological and pathological conditions, and across multiple cell types, albeit to different degrees. Fundamentally, irrespective of the source of membrane disruption, aberrant calcium influx is the common stimulus that activates the membrane repair response. Inadequate repair responses can tip the balance between physiology and pathology, highlighting the significance of plasma membrane integrity. For example, an over-activated repair response can promote cancer invasion, while the inability to efficiently repair membrane can drive neurodegeneration and muscular dystrophies. The interdisciplinary view explored here emphasises the widespread potential of targeting plasma membrane repair mechanisms for therapeutic purposes.

## Introduction

All mammalian cells are equipped with an evolutionary conserved membrane repair machinery that works to constantly ensure membrane integrity. Such widespread presence throughout the human body and across species highlights that the health of membranes is of uttermost importance for cell survival. Not only is the membrane repair response important for the maintenance of homoeostasis and “normal” cell functioning but failure to sustain membrane health can result in acute and chronic pathologies. This review assesses the role of membrane integrity and how it can be lost in different physiological and pathological contexts, including:

- skeletal muscle contraction and chronic myopathies;
- cardiac muscle contraction and acute injury (such as ischaemia–reperfusion injury);
- pore-mediated injuries triggered by immune cells and cytolysins;
- neuronal membrane injuries during physiological ageing and both acute and chronic illness (brain injury and neurodegenerative diseases, respectively); and
- migration-induced injuries in immune cells and fibroblasts under physiological conditions and in cancer cells during invasion and metastasis.

On the contrary, overactivation of the repair machinery has proved to be advantageous for cell survival. Cancer cells tend to overexpress proteins involved in the plasma membrane (PM) repair machinery for a robust repair response to its recurring injuries during cancer growth and invasion^1,2^.

Undoubtedly, cell life critically depends on the maintenance of PM integrity^1,3,4^. Beyond functioning a physical barrier between the extracellular and intracellular matrices, the PM is dynamic and highly responsive to these environments. It bridges intracellular signalling cascades and extracellular signals (e.g. ions, hormones, cytokines, enzymes and factors), allowing cellular communication with the surrounding environment^5^. Disruption of the PM, due to mechanical or biochemical stresses, poses an immediate threat to cell survival^1,6^. Furthermore, extensive dyshomeostasis can result in calcium toxicity, activation of proteolysis, osmotic stress and oxidative damage^6,7^, eventually leading to cell death. However, cells have adapted to be able to cope with membrane injuries by activating robust repair mechanisms^4^.

We^1^ and others^6–9^ have investigated and reviewed extensively the different known PM repair mechanisms available to cells, depending on the cell type and characteristics of the wound^1,10^. Here, we would like to stress that repair mechanisms are neither mutually exclusive nor restricted to certain cell types. In fact, the ability to perform biological processes that are vital for eukaryotic cell life appears to have co-evolved with the ability to repair membrane damage^6^. For example, endocytosis is an ancient and robust molecular event used in endocytosis-mediated membrane repair^11^. Other perspectives have also been explored: that dedicated repair mechanisms may not have developed per se and instead cells reused opportunistically several mechanisms dedicated to other tasks to cope with membrane disruptions^4^. Conversely, these repair mechanisms may have been established prior to other molecular machinery, as the first aim for a cell is not to perform non-essential membrane-remodelling events (e.g. exocytosis) but to survive by preserving its membrane integrity^4^. Ultimately, this intimate relationship between primitive cell functioning and membrane repair highlights the fundamental importance of maintenance of membrane integrity for the life of a cell.

## PM repair mechanisms

**Fig. 1:**
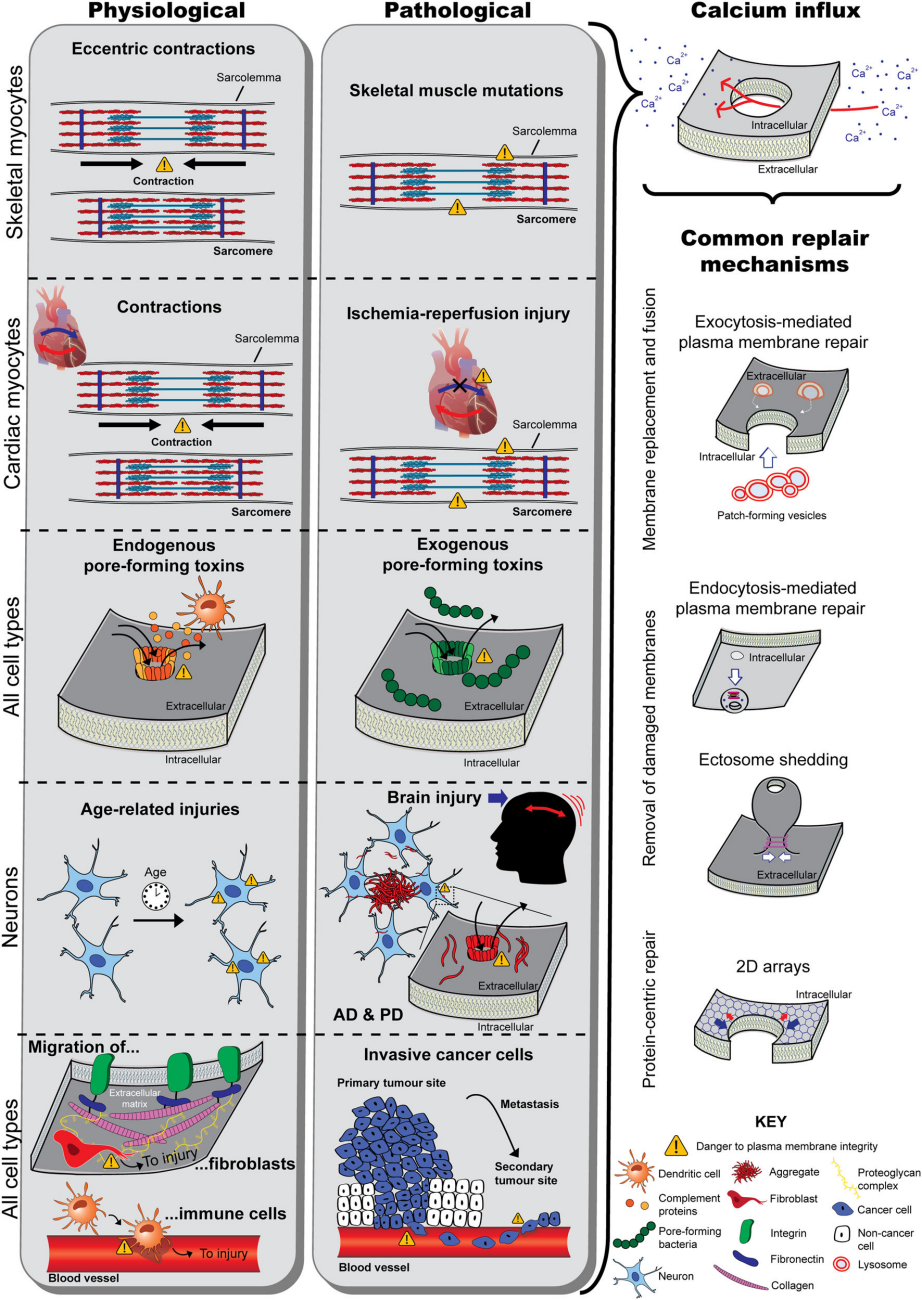
Schematic illustrating loss of membrane integrity under different physiological and pathological context and the common downstream events (calcium influx and membrane repair). Skeletal myocytes (first section of the grey boxes) undergo the danger of disrupting their sarcolemma during eccentric contractions under physiological conditions. Mutations in genes that produce fragile membranes and those that encode repair proteins are prone to membrane disruptions. Cardiac myocytes (second section) are also subjected to membrane disruptions during contraction, but during myocardial infarction the sarcolemma becomes subjected to injury. Pore-mediated injuries can affect all cell types. Under physiological conditions pores are formed by complement proteins produced by dendritic cells, as these arrange into the membrane attack complex. Under pathological contexts, toxins released from pore-forming bacteria damage membranes (third section). From a physiological background, age-dependent changes in the membranes of neurons drive loss of integrity. This phenomenon is also seen in brain injury and neurodegenerative diseases (such as Alzheimer’s disease and Parkinson’s disease) (fourth section). Cells that migrate through dense tissue also compromise their membranes. This includes fibroblasts and dendritic cells as they migrate through extracellular matrices and into blood vessels, as well as cancer cells during metastasis (fifth section). Irrespective of the source of damage, all injuries result in an abnormal influx of calcium into the intracellular space. This drives membrane repair mechanisms, which consist of membrane replacement and fusion strategies (e.g. exocytosis-mediated fusion of lysosomes with the damaged plasma membrane for patch repair or tension relief), removal of damaged membrane (including ectosome shedding or endocytosis-mediated repair), or protein-centric repair mechanisms (e.g. formation of 2D arrays by repair proteins to promote wound constriction).

Different cell-intrinsic PM repair mechanisms have been reported, including membrane fusion and replacement strategies (via exocytosis-mediated repair), removal of damaged membranes (by endocytosis-mediated repair or shedding), and protein-driven membrane remodelling and wound closure^1,7,8^ (Fig. 1).

In exocytosis-mediated repair, intracellular membrane sources (including endosomes^12^, enlargeosomes^13^, reserve/secretory granules^14,15^ or, more commonly, lysosomes^12,16–25^) fuse with the PM. Early studies were limited to endothelial cells^26^, fibroblasts^20,24–28^ and sea urchin eggs^14,27,29^, questioning the existence of this repair mechanism in other cell types. However, more recent studies have reported lysosome-mediated repair in muscle cells^23,30–33^, astrocytes^22^ and macrophages^21^. The fusion can occur at or close by to the PM wound site, for patch repair or tension relief, respectively. The former model results in a continuous membrane over the wound site^1,14,27^, while the tension-reduction model reduces tension in the wound area, thus bringing the membrane edges closer together to promote subsequent resealing^1,34^. Repair by patch formation is believed to resolve large wounds, while the tension relief mechanism may be activated by smaller wounds^7^. The underlying mechanism is triggered by molecular sensors that detect the injury and is followed by the nucleation of intracellular vesicles at the injury site and subsequent fusion^14,30^. Proteins involved include: SNARE proteins^14^, dysferlin^15,32,35^ and Annexin (ANX) A1^15,36^.

The literature remains controversial as to whether lysosome-exocytosis PM repair is a biologically relevant repair mechanism across cell types. Conceptually multiple questions remain unanswered (reviewed in ref. ^37^) and direct visualisation of lysosomal-exocytosis repair has only been recently achieved in *Xenopus* oocytes^15^. It is questionable whether injury-activated exocytosis of lysosomes (and other intracellular membrane sources) can meet the spatiotemporal needs required for efficient repair. Firstly, most lysosomes are perinuclear, thus injury sites at the cell periphery (e.g. cellular protrusions) are largely devoid of lysosomes^16,38^. Secondly, long-distance intracellular trafficking of bulky lysosomes from the perinuclear zone to the periphery is largely microtubular-based^39^, although there are some reports of actin-dependent, microtubule-independent transport^40^. These mechanisms of active transport are expected to be relative slow^16^, surpassing the second to sub-second window required for efficient repair^39,40^. Taken together, some argue that it is unlikely that a sufficient number of lysosomes will be locally available for PM repair^16,25^. These underlying reasons could explain why some studies argue that inhibition of lysosomal repair did not significantly affect the overall outcome of PM repair^16,41^. On the other hand, the opposite scenario has also been reported^17^ and both conventional lysosomes^18,42^ and peripheral (membrane proximal) lysosomes^12,25,43^ have been implicated in repair (although more recent studies reporting lysosomal-mediated repair favour the peripheral lysosomal hypothesis). Live-imaging-based studies report fusion of the endosomal/lysosomal compartment with the PM within seconds^26^ and 1–2 min^12^ post-injury, while smaller intracellular membrane sources that migrate faster^39,40^ and/or may be locally available in the cortical actin cytoskeleton (such as reserve/secretory granules) fuse in the second range^14,15^.

Not only is the population of lysosomes subject of much debate, the underlying mechanism of lysosomal-mediated repair has also been challenged. Andrews et al.^19^ cross-analysed recent and older studies that incorporated or lacked extracellular endocytic tracers in their experimental design, respectively, and investigated the existence of this repair mechanism. Recent studies observed that, in parallel to lysosome exocytosis, calcium influx also triggered a massive and rapid formation of endocytic vesicles at the injury site via invagination of the PM^11^. It was proposed that these small endosomes eventually merge to form larger ones and fuse with the PM before they rupture outwards (termed explodosis) to form a continuous barrier between the cytoplasm and the extracellular matrix, functioning as a large patch^15,19^. Given this observation and proposed mechanism of action, the authors argue that the “patch” hypothesis may have arisen from misinterpretation of these rapidly formed endocytic vesicles as the exocytic pre-existing intracellular compartments reported in earlier studies^19^. Of note, such endosome formation and fusion was observed in oocytes (<1 min post-injury)^15^. It remains unclear if this model translates to other cell types and occurs within the necessary timescale to be physiologically-relevant).

Less controversial is endocytosis-mediated PM repair. This repair mechanism removes small damaged membrane regions or pores from the PM by clathrin and caveolin-mediated endocytosis, to be subsequently sorted for degradation^7,12,44,45^. Another strategy for the removal of damaged membrane (such as membrane-containing pores) is microparticle/ectosome shedding^1,7,46^. This mechanism relies on the Endosomal Sorting Complex Required for Transport (ESCRT) machinery^7^.

Wound resealing can also be driven by inherent properties of repair proteins in a more protein-centric manner that does not rely on endocytic or membrane budding events^1,7^. The binding of repair protein to the sites of injury can promote wound closure in several ways. Firstly, in collaboration with actin cytoskeleton remodelling^35^, repair proteins can modulate the shape of the membranes at the wound periphery, inducing bending of lateral membranes, which promotes wound closure^47–50^. Secondly, such binding reduces lateral lipid tension, again promoting fusion of adjacent membranes. Thirdly, the self-aggregation properties of repair proteins enable the formation of 2D arrays around the wound perimeter, which also restricts wound expansion^1,7,31,51^. ANXs, including A4 (ref. ^52^), A5 (refs. ^31,51,53^) and A6 (ref. ^52^) have been shown to participate in PM repair of mammalian cells through these protein-driven mechanisms^1,49,51^, as well as dysferlin and mistugunin-53 (MG53)^7^. These mechanisms are thought to come into play in the repair of both small and large wounds^7,50,52^.

There is increasing evidence to suggest that different repair mechanisms occur in combination for an adequate repair response to be mounted. For example, lysosomal exocytosis occurs when microparticle shedding failed to eliminate toxin-formed pores^16^, and exocytosis-mediated repair in the aftermath of wound closure by 2D protein arrays^19,51^. The existence of multiple repair mechanisms with the same end-goal (i.e. restoration of PM integrity) points to functional redundancy in the repair response, again supporting its significance. Furthermore, not only do mechanisms co-operate during repair, the protein toolkit is also shared. The role of ANXs, S100 proteins, MG53, synaptotagmins, calpains and dysferlin across membrane repair mechanisms has been extensively reviewed^1–3,7^. The majority of these are calcium- and phospholipid-binding proteins, functioning as calcium sensors or regulated by calcium^1,3,48,49,54^. The role of a membrane repair proteins at an organ-level is largely dictated by its tissue expression pattern; however, it can influence other organs after secretion into the circulating system. MG53, for example, is specifically and highly expressed in skeletal and cardiac muscle, where it plays a critical role in maintaining muscle cell integrity. However, once secreted MG53 can help the repair of other organs and cell types, such as the kidney^55^, liver^56^, lungs^57^ and neurons^58^.

Regardless of the mechanism(s) employed by cells to respond to membrane injury or the source and type of injury, the key stimulus to initiate membrane repair is the same: a rise in intracellular calcium (Fig. 1). Since the PM partitions a ~10,000-fold calcium gradient, a transient disruption in membrane integrity results in an abrupt calcium influx^59^. The cell interprets this as an “immediate danger” signal, as it triggers degenerative biochemical and structural events that initiate death cascades^3^. Therefore, the cell has to rapidly reseal the wound. For example, a large PM disruption (with >1 µm diameter) results in local and rapid (in the sub-second to second timescale) Ca^2+^-activated homotypic vesicle fusion^36^. Calcium entry is considered essential for wound detection and repair and dictates the magnitude of PM repair response activated, as calcium entry is proportional to wound size and number^4,7^. The affinity of repair proteins for calcium influences which proteins are recruited to the injury site and this in turn shapes the downstream mechanisms elicited^7^. Furthermore, oxidation may also be an activator of membrane repair, since acute injury also causes transient changes in redox state near the site of injury^7,30,60^.

The need to seal a torn membrane and halt the uncontrollable influx of calcium from the extracellular environment to prevent cell death is clear. However, one can envision that PM repair is not complete after initial membrane resealing^15,19,61^. Instead, a process of “cell restructuring” follows to return the wound site to its original state, with the adequate repertoire of PM lipids and proteins and a fully restored cortical cytoskeleton, to ensure functionality^6,8,15,61^. All repair mechanism described could, in principle, contribute to membrane regeneration, with the exception of wound patching and the protein-centric repair mechanisms discussed, such as 2D protein arrays^8^. In fact, it has been reported that damaged portions of the membrane can be actively removed by endocytosis-mediated repair or exososomal shedding^6,9^, while exocytosis-mediated processes may replace the resident lipids and proteins lost at the site of injury^8^. Injury-induced lysosomal secretion of acid sphingomyelinase triggers the formation of endosomes that internalise the lesion^17^. While macrophage-mediated repair (phagocytosis) is a non-cell-autonomous mechanism for the removal of the repair patch^62^. Such remodelling phase of PM repair has been proposed to occur at 60–240 s post-injury^6,61^ (or may even take longer^8^), while membrane permeability is restored within 30 s of injury^6,9,61^. Therefore, as an initial response, mammalian cells might rely on the rapid lateral recruitment of membrane around the PM wound for resealing (or other strategies to halt calcium influx). Thereafter, membrane removal and replacement strategies (exocytosis- and endocytosis-mediated processes) are likely to become activated to restore adequate PM integrity and composition^61^.

PM disruptions have been documented under physiological conditions, particularly in mechanically and metabolically active tissues, such as skeletal muscle^6,7^. More differentiated cell types are particularly susceptible to damage, as they have limited self-renewal capacity^7^. Therefore, cell types that are terminally differentiated and those that experience frequent membrane injury rely strongly on PM repair mechanisms for survival^3,7^. Defects in the intrinsic membrane repair mechanisms have been linked to various disease states, including muscle dystrophies, heart failure and neurodegeneration^30^. Interestingly, Moe et al.^8^ proposed that membrane disturbances may not only result from defeats in PM resealing, but also from a compromised membrane regeneration potential in the aftermath of wound closure. Another question that remains unanswered is whether failure to repair or sustain adequate membrane integrity can tip the balance between homoeostasis and dyshomeostasis, driving from physiological to pathological states.

## Skeletal muscle injuries

### Physiological conditions

The most extensively studied model of PM injury has been skeletal muscle. Due to its elongated morphology and the forces of contraction the PM of myocytes (sarcolemma) is prone to lesions^63^. At basal conditions, the percentage of wounded myocytes in an adult rat model was reported to be 3.13%, while exposure to eccentric contractions increased PM lesions by 6.7-fold^64^. This is accompanied by an increase in membrane permeability, allowing the leakage of the large creatine phosphokinase protein. The repair mechanisms in place were responsible for the decrease in the percentage of wounded myocytes observed 24 h post-exercise to 5.63%, thus the majority of the wounds resealed promptly and survived^64^. Unrepaired membrane wounds can result in the aberrant release of growth factors due to increased membrane permeability (referred to as the “wound hormone hypothesis”), as well as necrosis and inflammation, driving fibre hypertrophy, degeneration and death^64,65^. Hence, PM disruption is an early form of structural damage in myocytes. Focal areas of fibre damage have been detected in humans following eccentric exercise and might represent membrane wounding^64^. In addition to mechanical stress, eccentric contractions also generate reactive oxygen species (ROS), which result in oxidation of membrane lipids, referred to as lipid peroxidation^66^. As discussed later, lipid peroxidation also contributes to abnormal membrane permeability.

### Pathological conditions

Muscular dystrophies (MDs) have been named to reflect the defective gene implicated (e.g. “laminopathies”, “titinopathies”, “dystrophinopathies”, “dysferlinopathies”, and so on)^67^. Mutations in these genes produce fragile membranes and compromise membrane repair, predisposing membranes to injury (whether due to mechanical stress or other stressors). The resultant PM instability is believed to contribute to dysregulated calcium homoeostasis and disease pathology^23,35,67–72^. Clinically, this group of muscle diseases are characterised by progressive weakness, atrophy and degeneration of skeletal muscle^67,69,73^. It is widely accepted that the early stage of the disease is characterised by increased membrane permeability^72,74^.

Among the 30 different inherited muscle diseases^67^ are Duchenne MD^32,73,75^, Becker MD^75^, Tibial MD^76^ and Limb-Girdle MD^77–81^, Miyoshi myopathy^31,32,73,78,81^ and Niemman-Pick Disease^69^. Heterozygous mutations in the sarcomeric protein titin cause Tibial MD, while homozygous mutations drive the more aggressive Limb-Girdle MD. Similarly, absence of dystrophin or expression of a non-functional protein cause Duchenne MD, while a reduction of wild-type dystrophin or expression of a partially functional protein cause Becker MD^67^. Both titin and dystrophin play structural roles in the sarcoplasm during muscle contraction and stretch, protecting from structural stresses^67^. Likewise, dyferlin links integrins with the cytoskeleton. When dysferlin is mutated a form of autosomal recessive inherited MD manifests either in proximal muscles (Limb-Girdle MD) or distal muscles (Miyoshi Myopathy)^74,78,81^. It is believed that these MDs are not solely caused by the disrupted cytoskeletal matrix, but due to the defective calcium-dependent, vesicle-mediated repair, given the role of dysferlin in repair^33,73^. Limb-Grindle MD can also be caused by mutations that affect transmembrane proteins called sarcoglycans^80^ or calpain-3 (ref. ^79^). The latter is predicted to cause defective membrane repair due to the absence of calpain-mediated cleavage of ANXA1 and ANXA2, which may be critical for patch formation and/or membrane insertion^33^. Of note, calcium-dependent calpain-mediated cleavage of dyferlin is also thought to mediate rapid vesicle fusion during membrane injury^82^. With regard to Niemman Pick sisease, both increased susceptible to injury and reduced resealing ability are hallmarks of the pathomechanism^69^. Firstly, the related mutation in the acid sphingomyelinase enzyme causes overaccumulation of sphingomyelin at the PM and lysosomes, which contributes to membrane instability^83^. Secondly, wound-triggered extracellular release of acid sphingomyelinase from lysosomes for endocytosis-mediated lesion removal is compromised^17^. The pathology is not muscle-specific, being associated with severe neurodegeneration.

Some MDs are idiopathic inflammatory myopathies (myositis), characterised by a chronic state of inflammation driven by abnormal membrane permeability that eventually results in the degeneration of muscle structure and function^84,85^. Once again, abnormal membrane resealing is believed to be an early event in these pathologies, resulting in increased exposure to intramuscular antigens, which activates the immune system. Autoantibodies targeting critical repair proteins (including anti-TRIM72/MG53 antibodies) may compromise membrane barrier function and promote progression of pathophysiology (establishing a feedback loop where decreased sarcolemma integrity promotes decreased resealing and increased antigen presentation). Thus, the autoimmune response triggered is primed to initiate a significant inflammatory response at the site of injury^84^. This was modelled in synaptotagim VII-deficient mouse models, which are defective in lysosomal exocytosis and resealing after wounding. Fibrosis, early inflammation and degeneration in skeletal muscle was observed^85^. Taken together, minimisation of PM disruption and efficient repair of membrane disruptions are essential for normal muscle function^63^.

### Membrane repair mechanisms

Skeletal muscle cells are reported to repair by mainly two active mechanisms: patch-mediated repair^30–32^ and cap-mediated repair (a protein-centric mechanism)^63,68^, in addition to local cytoskeletal remodelling^35,68^. Exocytosis of intracellular membranous structures (primarily lysosomes) to form a patch at the sarcolemmal wound has been reported^63,73,85^. Dysferlin has been proposed to act as a calcium-dependent “hook” between the membranous structures, allowing efficient fusion of the repair patch (labelled by MG53) with the sarcolemma^33,74^. Furthermore, the calcium-triggered interactions between dysferlin, ANXA1 and ANXA2 may be important in the aggregation and fusion of intracellular vesicles to the site of membrane injury^33,86^. Defects in dysferlin result in an abnormal distribution of ANXs^33^, an ineffective accumulation of intracellular vesicles at the membrane and a slower patch-mediated membrane repair^33,73^. On the other hand, cap-mediated repair is mediated by the sequential recruitment of actin and different ANXs (A1, A2, A5 and A6) to form a higher-order oligomeric structure (a tight “cap”) in a calcium-regulated manner that seals the membrane injury, restoring membrane integrity. Other repair proteins, including dysferlin, MG53, EHD1, EHD2 and BIN1, are also critical for repair and form the “shoulder” region of the cap^63^. In fact, upon MG53 and dysferlin recruitment to the site of injury (as early as 2^87^ and 10 s^74^ post-injury, respectively), the proteins form a lattice at exposed edges of the injury (probably bringing them together) and eventually fills the area of injury^74^. Moreover, MG53 is believed to act as a scaffold for the assembly of the membrane repair complex because it oligomerises rapidly once exposed to the extracellular mileu at the site of injury^74^. Given the redundant nature of the repair mechanisms, skeletal myocytes of MD patients, including dysferlinopathy and Duchenne muscular dystrophy, upregulate several repair proteins (including MG53, dysferlin and ANXA1) by two- to seven-fold^63^, in attempt to counteract the compromised membrane repair response.

## Cardiac muscle injuries

### Physiological conditions

The mechanical and metabolic struggles that cardiac myocytes confront with every heartbeat share similarities with that of skeletal muscle. Clarke et al.^65^ reported that an average of 25% of myocytes suffer from PM wounds at basal conditions; thus, transient and reparable wounding of cardiomyoctes is a constitutive event in vivo. As expected, wounding frequency increased approximately threefold after β-adrenergic stimulation of heart rate and force of contraction^65,86^. Likewise, the levels of cardiac-specific proteins detected in the blood increased by twofold after exercise^65^, supporting an increase in membrane permeability.

The left ventricle is exposed to the highest cardiac pressures even under physiological conditions and, over time, is more prone to develop hypertrophy and dysfunction^65,88^. Hence, membrane repair mechanisms become essential in older, rather than young and healthy cardiomyocytes^59^. Increased left ventricular mass is one of the adverse structural alterations that manifest in heart failure, in addition to cardiomyocte hypertrophy and inter-myocyte fibrosis^89^. With an ageing population, the degree of wear-and-tear of cardiac muscle throughout a lifetime increases, which in turn places the number of heart failure cases globally on an upward trajectory and drives its classification as an epidemic^89^. This supports the concept that PM-related disruptions in heart functioning can result from “normal” conditions, in physiological ageing.

### Pathological conditions

Mortality rates associated to heart failure are high and there is no cure for this common terminal illness^89^. Although heart failure is the long-term result of adverse structural alterations in response to pressure and volume overload, a range of cardiovascular diseases, including myocardial infarction, promote heart failure^89^. Acute myocardial infarction results in two types of myocardial damage: ischaemic injury and reperfusion injury, due to the initial loss and subsequent restoration of blood flow to cardiomyocytes. Ischaemia–reperfusion injury generates oxidative stress, resulting in lipid peroxidation that drives the breakdown of the sarcolemmal membrane and promotes inflammation and necrosis^6,89–91^.

At a cellular level, different mechanisms have been proposed to contribute to a poor membrane integrity, which is associated with increased membrane permeability. Firstly, ischaemia–reperfusion injury, by inducing apoptosis, results in loss of the asymmetric distribution of the PM phospholipids, having aminophospholipids (such as phosphatidylserine) exposed on the outer membrane leaflet^90^. Secondly, ATP depletion due to the lack of oxygen causes ATP-dependent pumps to fail, promoting ion dyshomeostasis and acidosis. The resultant osmotic stress drives cell swelling, while the calcium toxicity induces proteolysis and triggers mitochondrial dysfunction, production of ROS and apoptosis. Cell swelling, acidosis and oxidation compromise membrane properties and result in leaky membranes^6,92^. These cellular effects of ischaemia–reperfusion injury together with myocardial contractions exacerbate membrane injury and, if not repaired, initiate a viscous cycle of metabolic and mechanical stresses that promote necrotic events. Pathophysiological stressors, like ischaemia–reperfusion injury, exacerbate the need of membrane repair mechanisms in cardiomyocytes^59^. Moreover, mutations that render myocytes more susceptible to injury^86^ and defects in the membrane repair response can contribute to the progression of muscular dystrophies^30,71,86,88^.

### Membrane repair mechanisms

Despite the partial loss of membrane permeability and the ion homoeostasis, the heart continues to beat and with increased force, which suggests that cardiac myocytes can withstand large, but transient, stresses without permanent functional or electrical compromise^65^. This is due to the mechanisms in place that restore ion homoeostasis and revert death signalling cascades that might have initiated, as well as the membrane repair mechanisms that halt further toxicity. Unrepaired cardiomyocyte membrane injury causes irreplaceable cell loss, leading to fibrosis and, eventually, heart failure^59^. This highlights the importance of preventing loss of sarcolemmal viability and the vital role of the PM repair response. Although the molecular mechanisms of cardiac membrane repair are largely unknown, patch-mediated repair has been suggested^30,65^. Repair proteins involved include MG53 (refs. ^30,59^), dysferlin^30,59,71,86^, GRAF1 (ref. ^71^) and ANXs. Of interest, ANXs A2, A4, A5 and A6 have been reported to be upregulated in the failing human heart^93,94^ and may play a role in cardiac remodelling (fibrosis) and calcium handling^93^ and have anti-inflammatory and anti-apoptotic functions^90^.

## Pore-forming injuries

### Physiological conditions

Membrane disruption by pore formation is a strategy employed by the immune system under both physiological and pathological conditions, for immune surveillance and removal of foreign or damaged cells (such as tumour cells). The innate and adaptive immune systems rely on the complement system to remove “non-self” cells via complement-dependent cytotoxicity^95^. Here, a membrane attack complex assembles at cell surfaces and transmits the cell death signals^96^. The complex damages the membrane barrier, resulting in elevated intracellular concentrations of calcium ions and ROS and, eventually, cell swelling and necrosis^96,97^.

### Pathological conditions

In contrast to this endogenous pore-forming phenomenon driven by the immune system, exogenous pore-forming toxins released by pathogens result in pathological conditions^98^. Bacteria release toxins to counteract the host defence mechanisms (through phagocyte intoxication) and, eventually, drive loss of the host cell function. By controlling host–pathogen interaction, there is a window of time and opportunity for bacterial growth and establishment within the host^99^. Spreading to sterile regions can be problematic^100^. For example, the bacteria *Steptococcus pneumoniae* can spread to the respiratory tract where its toxin, Pneumolysin, disrupts the membranes of epithelial and endothelial barriers, causing life-threatening diseases including pneumonia, meningtitis and septicaemia^100,101^.

The pathomechanism of all cytolysins (including Streptolysin O, Perfringolysin O and Intermedilysin, Listeriolysin O and Pneumolysin) starts with its binding to the host’s PM. Most cholesterol-dependent toxins access the PM by binding to cholesterol. At the membrane, the toxins oligomerise into large, ring-shaped pre-pores of about 30–50 nm in diameter. Alpha-toxins form smaller pores (~2 nm)^10,98^. The complex then undergoes a conformational change that perforates the membrane, forming stable transmembrane pores^98,101^. As a result, the membrane becomes permeable to ions, metabolites and proteins, which dramatically disrupts cellular homoeostasis^10^. At sub-lytic concentrations of Pneumolysin, the intracellular concentration of calcium ions in the majority of permeabilized cells was reported to increase from ~100 nm to 2–10 µM, while at lytic concentrations this was within the 10–20 µM range^101^. The extent of calcium influx correlates with cellular fate, activating programmed cell death pathways at sub-lytic levels and uncontrolled necrotic cell death and lytic levels^10,101^. Other than the PM, intracellular membranes (such as the mitochondrial and lysosomal membranes) can also become injured by pore-forming toxins^10^. Overall, toxicity depends on the stoichiometry and size of active pores and the ability of the cell to neutralise the pores via its membrane repair mechanisms^10,54,101^.

### Membrane repair mechanisms

The detrimental effects of pore-forming toxins can be prevented by PM repair^101^. The most common repair mechanism reported for this type of injury is microparticle shedding (exocytosis-mediated), although patch-mediated repair, engulfment (endocytosis-mediated) and blebbing have been described^98^.

Irrespective of the cause of pore-forming injury (whether endogenously or exogenously driven), cells can rapidly eliminate the pore directly from the PM by shedding of vesicles, a process referred to as exo-vesiculation, ectocytosis or microparticle shedding^10,97,98,102^. This causes a reduction in intracellular calcium levels and promotes cell recovery^102^. It has been proposed that blebbing proceeds the shedding of microvesicles^10,98,102^. The formation of blebs (cytoplasmic spherical protrusions that are connected to the cell body by a thin neck) not only isolates the pore but also confines calcium ions, protecting the cell from calcium toxicity and loss of cytosolic content^10^. However, blebs have also been shown to retract, rather than shed, which could suggest a role as “clogging” structures^10^.

Alternatively, pores can be inactivated or internalised by patch formation or endocytosis, respectively. When internalised, pores accumulate in the endocytic recycling compartment and becoming packed within multivesciular bodies or subjected to degradation^10,97,98^. In patch-mediated repair, the excessive membrane permeability is counteracted by the exocytosis and fusion of intracellular membranous structures^98^, such as the lysosome^101^. Exocytosis-mediated repair to decrease PM tension, promoting spontaneous resealing or active repair by other mechanisms (such as blebbing), has also been implicated in the repair of pore-mediated injuries^10^.

It is generally accepted that cells repair from pores in their PM by microparticle shedding^16,54,101,102^, patch formation^98^ and/or endocytosis-mediated removal of the pores^11,98^. ANXs A1 (refs. ^54,102^), A2 (refs. ^101^) and A6 (ref. ^54,102^) have been implicated in the shedding of SLO pores and their recruitment to the damaged PM is sequential (dependent on their Ca^2+^ sensitivities), ensuring continuous fine tuning of the repair response mounted^54,101,102^. Microparticle shedding may be optimal for an early elimination of toxin pores, while lysosomal fusion may occur at a later stage and be best suited for the repair of secondary injuries^16,101^. Finally, endocytosis-mediated removal of pores has been described as occurring both in the immediate response to the influx of calcium^11^ and in the aftermath of membrane resealing, during restructuring^98,103^. It is highly likely that the coordinated action of many mechanisms protects against intoxication by various pores^10,98^. Nevertheless, PM repair mechanisms are unable to prevent irreversible cell damage if the intracellular calcium concentration rises above this critical threshold (10 µM)^102^.

## Neuronal injuries

### Physiological conditions

Although all mammalian cells evolved with an intricate membrane repair system, the concept of neuronal injury at basal conditions has not been directly addressed. Nonetheless, a study focusing on the effect of pathological stressors on neuronal membrane integrity documented that an average of 13.88% of the cortical neurons in an adult rat model show membrane disruptions at basal conditions^104^. Thus, membrane injuries are sporadic but present at physiological conditions.

These membrane disruptions are likely to be more relevant during physiological ageing. There is an age-associated increase in membrane unsaturation, making membranes increasingly susceptible to lipid peroxidation because polyunsaturated fatty acids are extremely vulnerable to oxidation^105,106^. Membrane oxidation results in increased membrane rigidity, decreased thickness, increased permeability and loss of function^105–109^. Interestingly, this age-related decline in membrane health could help to explain why age is the greatest risk factor for neurodegenerative diseases.

The fact that the vast majority of neurons are terminally differentiated makes this cell type more susceptible to membrane injury than others with greater plasticity and self-renewal capacity^7^. Therefore, an adequate membrane repair response is essential to maintain neuronal health. The inability to repair damaged membranes is believed to be one of the key mechanisms underlying progressive and severe neuronal degeneration^110^.

### Pathological conditions: brain injury

The pathomechanism of traumatic brain injury (TBI) is largely initiated by a breach in neuronal membrane integrity. TBI is estimated to affect 235 per 100,000 people annually in Europe^111^ and about 2.8 million people in the US^112^. TBI (and spinal cord injury) is characterised by two mechanisms of damage: the primary mechanical injury (focal) and secondary injury (diffuse) mediated by multiple processes^104,113^.

Firstly, the rapid increase in axonal strain causes transient membrane tearing (termed mechanoporation)^114^ within seconds of injury^104^. Such mechanical insult can surpass structural thresholds and result in cell death. However, there is a growing appreciation that the sub-lethal forces lead to a “pathological cascade” of events that last several hours after the initial injury and severely impair neuronal functioning^115^. Such neuronal cell degeneration after physical damage is driven by disruptions of the intracellular environment, which in turn are triggered by neuronal membrane damage^110^.

Damaged neurons form focal axonal swellings within 2 h post-injury and these increase in size eventually causing axolemmal disconnection at 4–6 h post-injury (referred to as secondary axotomy)^115^, which may persist over days and even weeks^116^. These axonal swellings are driven by focal disruptions of axonal transport, which causes the accumulation proteins, such as β-amyloid precursor protein (β-APP), and organelles at such foci^115,116^. Intra-axonal cytoskeletal abnormalities also result in compaction of neurofilaments within injured axons and neurofilament-rich inclusions have deleterious effects on neuronal function and survival^115^.

The two consecutive waves of membrane disruptions (the initial mechanoporation and secondary axotomy) result in a rapid and sustained loss of homoeostasis that aggravates neuronal injury. The increase in membrane permeability can be detected within 5 min of damage, peaks at 1–6 h post-injury and persist until 72 h^117^. It causes an uncontrolled influx of calcium, activating calpain-induced proteolysis and calcium-dependent phospholipases, and promotes lipid peroxidation, which further damages membranes^115^. The loss of ion homoeostasis abolishes the ionic gradients necessary to sustain neuronal electrical activity and alters neurotransmitter release^115,118^. Overall, these events lead to depolarisation, altered metabolism, impaired connectivity, cellular swelling, inflammation and cellular degeneration^115,116^.

### Pathological conditions: neurodegenerative diseases

Acute brain injury can lead to chronic and progressive neurodegeneration^114,118^. It is well established that TBI and other physical injuries are major epidemiological risk factors for dementias including Alzheimer’s disease (AD)^115^. At a molecular level, in the aftershock of the acute injury, brains tend to accumulate disease proteins^114^, including amyloid-β protein (Aβ)^119,120^, hyperphosphorylated tau^121^ and neurofibrillary tangles^121^.

AD and Parkinson’s disease (PD) are the two most common neurodegenerative diseases and are clinically characterised by memory loss and motor control impairments, respectively, in addition to cognitive decline^122,123^. In AD, the disease protein Aβ is cleaved from a large precursor, APP, and accumulates as diffuse (non-fibrillar) or fibrillar plaques in dystrophic neurites^123^. In PD, the disease protein is α-synuclein (α-syn) and it also accumulates to form aggregates, named Lewy bodies and Lewy neurites^122^. Cellular toxicity has been attributed to all forms of Aβ and α-syn (monomeric, oligomeric and fibrillar), albeit to different degrees, and the oligomeric forms are regarded as the most toxic species^122,124^.

**Fig. 2:**
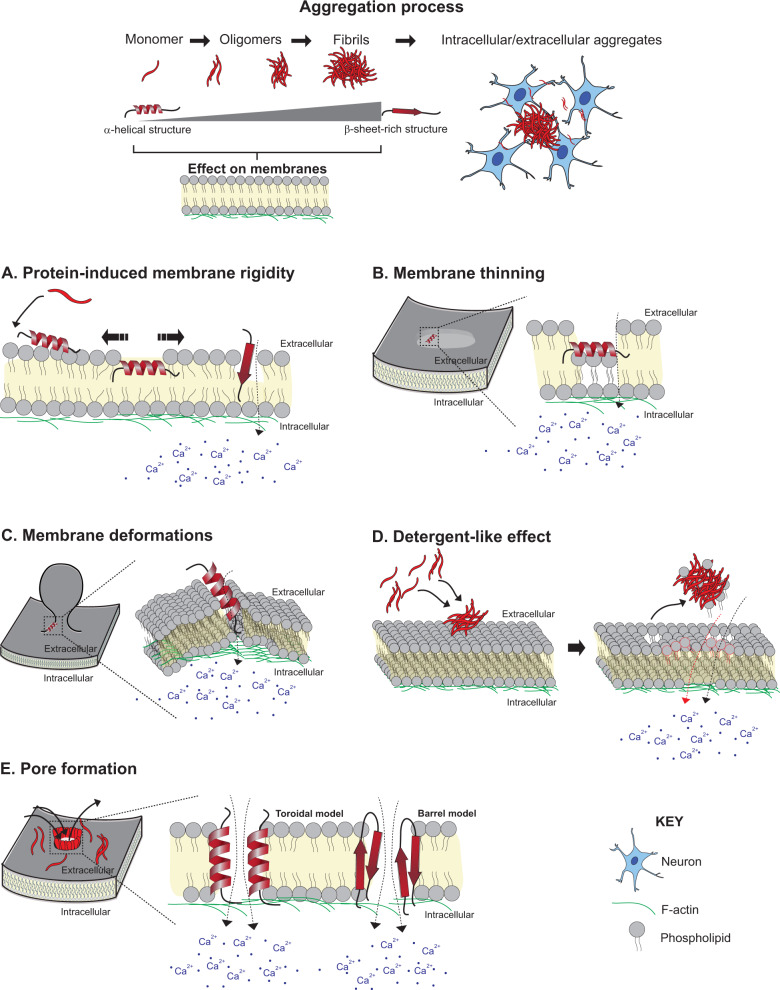
Loss of membrane integrity in neurodegenerative diseases. In Alzheimer’s disease and Parkinson’s disease, the disease proteins amyloid-β and α-synuclein, respectively, aggregate from monomers to aggregates, transitioning from intrinsically unfolded or α-helical structures to β-sheet-rich structures. Different mechanisms of protein-induced loss of membrane integrity are represented. **a** Protein–lipid interactions and lipid-mediated conformational changes result in protein incorporation into the membrane. This increases surface pressure and membrane rigidity, and can promote membrane thinning and deformation. **b**, **c** Proteins may induce lipids to reposition out of the plane of the membrane, resulting in membrane thinning (**b**) and curvature (**c**). **d** During aggregation, oligomers extract phospholipids from the bilayer and incorporate into the growing fibrils, causing membrane rupture. **e** Amyloidogenic proteins form pores in membranes. α-Syn forms pores rich in α-helical or β-sheet structures (toroidal and barrel models, respectively). All these mechanisms result in an influx of calcium from the extracellular environment and an efflux of cytosolic content.

Loss of PM integrity is also a pathomechanism of AD and PD, and most likely other proteinopathies. Membrane lipids play an important role in the kinetics of Aβ and α-syn aggregation and, in turn, resultant toxicity^125–127^. Lipids influence the aggregation process through lipid-induced conformational changes^122,126,128,129^ or mass action^130,131^ (i.e. local confinement of proteins to a small or 2D surface (e.g. the membrane of a vesicle), increasing the probability of inter-molecular interactions and driving aggregation^130,131^). Conversely, disease proteins can cause membrane permeability by different actions^131^: protein-induced membrane rigidity^119,132^, membrane thinning^133,134^ and deformations^133–137^, as well as detergent-like effect^119,129,138^ and pore formation^122,124,128,132,139,140^ (Fig. 2). In parallel, cells in degenerating brain regions need to cope with the increasing oxidative stress, which itself damages membranes through lipid peroxidation^106,108,127,141^, as discussed.

By inserting into membranes, disease proteins can induce membrane stiffness (either due to their rigid β-sheet-rich structure or a protein-induced increase in global ordering of membrane lipids). To relieve the excess surface pressure that inserted proteins impose, membrane lipids can remodel by repositioning out of the membrane plane, which results in membrane thinning or curvature^133,134^. All these insults can increase membrane permeability^132,133^. Excessive membrane curvature can also cause organellar deformations and dysfunction (including Golgi fragmentation and damage to lysosomes, mitochondria and vesicles). This phenomenon has been reported for lipid-bound helical α-syn^129,134,137^ and Aβ oligomers^138^. It may be the most prominent pathomechanism of the E46K α-syn mutant associated with the severe familiar PD. Modification of the amphiphatic nature of its membrane-binding helix transformed α-syn from a curvature sensor to a robust curvature inducer^135,136,142^.

Amyloidogenic proteins can also deleteriously affect membranes through a detergent-like effect. During the process of aggregation, oligomers induce widespread lipid extraction from the bilayer and subsequently incorporation into the growing fibrils. Over time this can rupture membranes^129,138^. The degree of these protein-induced membrane effects correlates with the concentration of the membrane-bound protein^125,129,134^. Therefore, loss of membrane integrity could help to explain the dose-dependent effect of disease protein steady-state levels on disease severity, as observed for familial α-synucleinopathies (i.e. gene dosage effect)^143^.

The membrane perturbation event likely to result in greatest immediate cytotoxicity and loss of homoeostasis is the formation of pores. Such amyloid pore hypothesis has been described for α-syn and Aβ^124,139,140,144,145^. The resultant calcium influx is comparable to that generated by pore-forming toxins^124^. The diameter of pores created by these disease proteins lies between ∼1 and 2.5 nm^122,139^. Well-ordered, oligomeric membrane-spanning pores are formed in a defined manner. Firstly, monomeric α-syn accumulates at the membrane by mass action^130^ or oligomeric α-syn^122^ or Aβ^126,128^ species translocate to the membrane and orient their hydrophobic residues towards the hydrophobic core of the lipid bilayer. Once embedded, a ring-like structure with a central hole is formed. For Aβ oligomers, U-shaped β-sheet structures have been reported to be capable of pore formation^128^, while for α-syn pores with both α-helical structures (formed by the sequential binding of monomeric α-syn) and β-sheet-rich structures have been reported and named toroidal and barrel models, respectively (represented schematically in Fig. 2e)^138^, although β-sheet oligomers/fibrils are believed to be more toxic^128,138–140,144^. Membrane-mediated toxicity of these proteinopathies can be mediated by a combination of mechanisms (e.g. pore formation and curvature-induced membrane fragmentation)^128,132^.

### Membrane repair mechanisms

Loss of neuronal membrane integrity is a common feature across pathological conditions (including traumatic brain/spinal cord injury and neurodegenerative diseases like AD and PD), but is also present, to a lesser extent, under physiological conditions. However, the membrane repair mechanisms activated in response to these PM injuries remain largely unknown and understudied. Some groups pioneered in this field by investigating possible repair proteins involved, and these findings will now be reviewed. A step forward would be to elucidate the underlying mechanisms in which these repair proteins are involved.

With regard to brain injury, although increased membrane permeability correlated with cell death in models of TBI, some cells that were initially damaged were capable of restoring their plasmalemma integrity and, consequently, did not progress to cell death^104,117^. However, the membrane repair response employed is unknown. A role for ANXs has been hypothesised based on the findings from a model of spinal cord injury, where the expression of ANXs A1, A2 and A5 correlated with repair kinetics and localised close to the site of injury^113^.

Protein-driven repair has also been proposed for chronic pathological conditions, such as neurodegeneration^146,147^, although the supporting evidence is still limited to the pioneering studies. Eberhard et al.^146^ found that across all acute central nervous system damage and chronic degeneration pathologies assessed (inflammatory diseases, infarcts, seizure disorders and AD), ANXA6 had altered subcellular distribution in affected neurons and that the expression of ANXs A1, A2 and A4 was increased in reactive astrocytes. In AD, specifically, ANXA6 was found within granulovacuolar bodies in degenerating pyramidal neurons and associated with neuronal cell membranes, whereas ANXA2 was expressed primarily in the PM of reactive astrocytes associated with β-amyloid plaques^146^. Interestingly, ANXA2 and S100-A10 heterotetramer complexes primarily associated with the PM of these glial cells^146^, pointing to the possibility of a collaborative repair mechanism mediated by these two repair proteins, which has been reported in PM repair by polymerisation of cortical F-actin and excision of the damaged membrane^47^. It has been hypothesised that the change in expression pattern of ANXs may represent a neural tissue response to limit damage, by promoting neuronal survival or recovery from injury, suggesting their involvement in neuronal and glial responses to acute and chronic injury^146^.

Dysferlin is another repair protein that has been implicated in PM repair, more specifically in exocytosis and endocytosis-mediated mechanisms^7,36^. Interestingly, the expression of dysferlin correlates with disease progression. While, little to no dysferlin neuronal staining was detected in control cases, it progressively accumulated in dystrophic neurites in AD brains in a manner that was proportional to disease severity (from mild to advanced cases)^123^. The deposition of dysferlin may be related to the inability of neurons to repair damage due to Aβ pathology^123^, although we lack direct mechanistic insight. Dysferlin may aid the membrane repair response through its calcium-dependent activity in the regulation of vesicle trafficking and membrane fusion^123^. However, seeing that insoluble and aggregated dysferlin was also detected in AD cases^123^ (in line with the formation of insoluble inclusions that is characteristic of proteinopathies^148^), one can envision that, during the course of disease, dysferlin (and other repair proteins) might become sequestered and unable to assist in membrane repair.

## Migration-induced injuries

### Physiological conditions

Cell motility is essential for multiple biological process. Dendritic cells and other immune cells, for example, must migrate and invade through tissue to reach the site of injury^149^, while fibroblasts are recruited to heal skin wounds^150^. Such mechanical activity of cells and the stress imposed on them when migrating through dense extracellular environment is a constant source of PM injury that occurs under physiological conditions^149–151^ (illustrated in Fig. 1).

A fundamental event during inflammation is leucocyte migration through vascular walls. The vascular basement membrane is a tightly packed network composed of extracellular matrix proteins (primarily collagen and laminins) and glycoproteins, posing as a formidable barrier for leucocyte migration. While the more invasive morphology of monocytes enables them to penetrate the vascular basement membrane by “squeezing” their cell bodies through, neutrophils impose greater tension on the vascular wall when migrating (transmigrated neutrophils carry basement membrane-derived fragments)^152^ and are likely to become injured in the process. It has been documented that dendritic cells deform their nucleus during migration, which transiently compromises the nucleo-cytoplasmic barrier^149^. To survive, migratory cells must rely on membrane repair mechanisms. The ESCRT III complex has been reported to reseal nuclear envelope ruptures in human dendritic cells^149^.

### Pathological conditions

Such phenomenon of migration-induced membrane injuries has been given more attention in the context of invasive cancer cells. During metastasis, cancer cells invade into the lymphatics and blood vessels by migrating through the basement membrane and dense interstitial tissue (depicted in Fig. 1). Despite the secretion of proteases and the alteration in the cytoskeletal architecture of cells to facilitate migration, the tremendous physical stress imposed can disrupt membranes. The process is then repeated when the tumour cells extravasate to reach a secondary site. The success of the invasion-metastasis cascade is key for cancer cells to spread from their primary site and correlates with poor patient prognosis^153–155^. To cope with the increased frequency of membrane lesions, invasive cancer cells tend to upregulate PM repair mechanisms^47,156,157^. Furthermore, during the course of malignant transformation, the PM reduces in stiffness by fivefold to accommodate the remodelling necessary for invasion^153^. However, such instability renders membranes prone to stretch-induced membrane pores^47,153^.

In attempt to quantify the extent to which aggressive cancer cells are predisposed to PM damage, MCF7 breast cancer cells of an invasive phenotype (ectopically expressing a truncated form of the oncogene ErbB2/HER2 that mimics the constitutively active form) were fivefold more likely to present signs of membrane damage under Ca^2+^-free condition as compared to their less invasive counterparts. This supports the hypothesis that increased motility and invasiveness correlate with enhanced membrane damage^2,47^.

The membrane homoeostasis of cancer cells is further compromised by their enhanced oxidative stress, which can also lead to lesions^2^. To support the rapid proliferation of tumours, cancer cells heighten their requirement for ATP, becoming more metabolically active, which is associated with increased ROS production. This triggers a viscous cycle, where ROS-induced mitochondrial damage further generates ROS^158^. Sources of ROS in cancer cells include stimulation of oncogenes, abnormal metabolism, hypoxia and aggravated inflammatory activities^159,160^. Therefore, a pro-oxidant micro-environment arises during tumour formation^160^. Such increased oxidative stress results in lipid peroxidation^159^, which deleteriously affects membrane integrity. However, the enhanced PM repair response of cancer cells counteracts these insults and the sub-lethal levels of oxidative stress can be used to favour cancer progression (growth and the acquisition of a malignant phenotype through ROS-induced DNA damage)^158,160^. Paradoxically, excessive ROS levels can overwhelm the repair mechanisms, resulting in cell death^158^.

### Membrane repair mechanisms

Several PM repair mechanisms have been implicated in the response to migration-induced injuries in physiological and pathological contexts, including exocytosis-mediated repair^47^, removal of damaged membrane (including excision^161^) and protein-driven membrane remodelling and wound closure^52^, in addition of cytoskeletal remodelling^2,47,151,153^.

The progressive and elaborate strategies that cancer cells adopt to respond to PM injuries during migration and invasion highlight the importance of these repair mechanisms on dictating cancer cell survival and progression. It has been proposed that as a first response, ANXA4 and ANXA6 become recruited to the wound to form a putative repair cap and promote resealing through induction of curvature and contraction forces, respectively, at the wound edges. In vitro, wound closure using this mechanism has been shown to occur within 10–15 s^52^. Another mechanism proposed to halt excessive membrane permeability in invasive cancer cells is by ANXA2-S100A11-mediated repair. Upon calcium influx, ANXA2 and S100A11 co-accumulate in a mutually dependent manner at the site of PM repair (within 15–45 s). As a heterotetramer, these proteins promote the aggregation and fusion of membranes at the site of injury^47^. In the aftermath of wound closure cancer cells have been found to restore membrane integrity through removal of damaged membranes^61^. In parallel to the recruitment of other repair proteins, ANXA1 is recruited in a calcium-dependent, S100A11-independent manner within 10 s of wounding and accumulates directly at the site of injury, where it functions to promote excision of the damaged cell membrane^47^. Another toolkit used to promote excision of the damaged membrane is ANXA7 and the ESCRT III complex. Upon calcium influx, ANXA7 interacts with apoptosis linked gene-2, facilitating proper ESCRT III complex recruitment and binding to the damaged membrane for subsequent shedding as ectosomes^161^. Importantly, multiple repair proteins, such as ANXA2, can manipulate cortical actin polymerisation to assist the closure of the wound^2,151^. Undoubtedly, the collaborative and cooperative nature of the different repair mechanisms enables cancer cells to promptly respond to the increased frequency of membrane injuries during invasion.

Commonly, the expression of repair proteins is altered during tumour growth and progression. For example, ANXA1 has been reported to be overexpressed in some tumour types, including oesophageal adenocarcinoma^162^, pancreatic adenocarcinoma^163^ and hairy cell leukaemia^162^, while ANXA2 is upregulated in breast cancer^164^, high-grade gliomas^165^ and kidney cancer^166^ (reviewed in ref. ^167^). Likewise, kidney tumours have shown increased dysferlin protein expression^168^. Increased expression tends to correlate with increased metastatic potential, tumour stage and poor prognosis^2,47,167,169^, which probably reflects the increased need for repair during invasion and metastasis, highly stressful events for the maintenance of membrane integrity^47,151^. However, the expression profile of repair proteins is tumour type-specific. In certain cancers, ANXA1 (refs. ^162,167,170^), ANXA2 (refs. ^167,171,172^), dysferlin^168^ and MG53 (refs. ^173,174^) have been ascribed as tumour suppressors. Increasing mechanistic insight into the non-canonical roles of repair proteins in tumour function could help to explain the discrepancies between tumour type and expression profiles^167^.

Although the underlying mechanisms of membrane repair have been extensively studied in the context of cancer, some of the same repair proteins have been found to be recruited upon physical stress in both cancer cells and non-cancer cells (such as immune cells), pointing to redundancy in the repair response. Namely, the ESCRT III complex not only repairs PM disruptions^161^ but also heals nuclear envelope injuries that result from migration-induced nuclear deformations in dendritic cells^149^ and healthy fibroblasts^154^, as well as fibrosarcoma and breast cancer cells^154^. The timely nucleo-cytoplasmic re-compartmentalisation that the ESCRT III complex offers upon nuclear envelope openings enables the vast majority of cells (>90%) to survive even repeated injuries^150^. This illustrates the robustness of the repair system and its recurrent need to ensure cell survival. Nonetheless, loss of nuclear envelope integrity has been linked to the normal ageing process and a variety of human diseases^150^, being both a physiological and pathological phenomenon.

## Conclusion

Membrane injury is a phenomenon that occurs frequently under both physiological and pathological conditions and across various cell types. Its restoration is key for cell function and survival; hence, cells are equipped with a robust, redundant and conserved repair toolkit that becomes activated and is largely irrespective of injury and cell type. While an adequate membrane repair response can prevent the initiation and progression of pathologies, sustained loss of membrane integrity may not only be a driver of physiological ageing, but also a common primary mechanism of pathogenesis across diseases. Whether cells are too efficient at repairing membrane injuries (in the case of cancer, specifically) or cannot adequately cope with injuries, maintenance of membrane homoeostasis is intimately connected to cell health and disease.

We have witnessed that a better understanding of the process of membrane repair in one cell type translates to others (with regard to stimulus, proteins and/or mechanisms involved). Likewise, this translational approach may foster the identification of therapeutic strategies for distinct tissues that are frequently subjected to membrane injury (both under different pathological conditions and in physiological ageing).

Boosting membrane repair may be a major therapeutic route for diseases related to poor membrane integrity, such as muscle dystrophies and neurons^1,7,110^. Different therapeutic strategies have been proposed (the use of membrane stabilising agents and exogenous expression of several recombinant proteins) and explored to different extents (from preliminary studies to clinical trials), although this is a recent and fast-growing field with wide-ranging potential. The membrane-stabilising agent, poloxamer 188, is FDA-approved multiblock copolymer surfactant^175,176^. It has been shown to seal stable defects in cell membranes of various cell types (including endothelial cells^176^, skeletal muscle cells^177^ and neurons^175^) caused by different types of injury (including ischaemia–reperfusion injury^175,177^ and mechanical-induced damage^176^). Due to its amphiphilic nature, the polymer can reversibly insert into the lipid bilayer (specifically at structurally disrupted membrane portions), forcing lipid molecules to pack tightly^178^ and re-establish the barrier function of the membrane^175,176,178^. Polymers seem to have medical utility across diseases (reviewed in ref. ^179^), including brain injury and cardiovascular disease^176^. Similarly, steroids have also been considered as membrane-stabilising agents. By integrating into damaged membranes, Vamorolone has been shown to stabilise the sarcolemma of dysferlin-deficient muscle cell and improve membrane repair of the myocytes following eccentric contraction-induced injury. In parallel, the anti-inflammatory actions of the steroids have been associated with reduced lipid peroxidation. Together, its actions may be beneficial in muscle dystrophies and brain injuries^180^. There is also evidence of improved membrane homoeostasis with the addition of proteins that aid repair. Acute treatment of acid sphingomyelinase promoted membrane repair in dysferlinopathic myofibres by rescuing the absence of injury-triggered secretion acid sphingomyelinase in this condition^181^. With regard to extracellularly added repair proteins, human recombinant MG53 has been shown to restore membrane integrity in a dose-dependent manner in both muscle and non-muscle cells^182^. MG53 treatment improved cardiac and skeletal membrane repair, ameliorated cardiomyopathy and the pathology associated with skeletal muscular dystrophy^6,30,59,91,182^. In contrast to its intracellular mode of action, extracellularly added MG53 may function by binding to exposed phosphatidylserine at the injured cell surface to facilitate repair and prevent the pro-inflammatory cascades associated^182^. Also, exogenous expression of ANXA6 had therapeutic benefit in muscle fibres, either by enhancing membrane repair and/or by reducing the susceptibility to injury through stabilisation of the PM^68^. Another therapeutic strategy with the same goal would be to activate endogenous repair proteins^3^—a largely unexplored avenue. However, this would require further understanding of the regulation of endogenous repair proteins by, for example, post-translational modifications, in order to activate/inhibit key enzymatic cascades by small molecules or antibodies.

Conversely, since the robust PM repair response of invasive cancer cells confers survival advantage, blocking repair mechanisms has promising therapeutic potential^1,7^. However, given the functional redundancy across repair proteins (such the ANX family of proteins) and repair mechanisms, it may be challenging to effectively diminish the repair capacity of cells^1^. Efforts to disrupt the interaction between ANXA2 and S100 proteins are underway^183^ and might compromise the invasive ability of breast cancer cells^47^. Alternative therapeutic routes could be to enhance the oxidation state of the PM of cancer cells specifically to exacerbate lipid peroxidation-induced injuries^184^, and to inhibit repair mechanisms associated with migration-induced nuclear injuries (e.g. via inhibition of the ESCRT III complex)^150^. Naturally, these strategies would have to be cancer cell specific to limit toxicity. Another strategy explored is the use of lectins. Lectins are multimeric protein or glycoprotein molecules capable of aggregating at the cell surface^185^. This action increases membrane permeability and free cytoplasmic calcium^185^. Furthermore, cell surface-bound lectins were found to potently inhibit PM repair by inhibiting exocytosis, becoming toxic to wounded cells^186^.

The link between maintenance of membrane integrity and overall tissue health has been previously unappreciated. Seeing that the initiation and progression of various human pathologies is characterised by membrane injury, modulation of membrane repair mechanisms holds tremendous therapeutic potential.
